# Supplementary material for: Malfunctioning CD106-positive, short-term hematopoietic stem cells trigger diabetic neuropathy in mice by cell fusion
Source: Commun Biol. 2021 May 14;4:575. doi: 10.1038/s42003-021-02082-5 (PMC8121918; doi:10.1038/s42003-021-02082-5)
Supplement: Supplementary file 2 — Supplementary information [file 42003_2021_2082_MOESM2_ESM.pdf]

Supplementary information

Malfunctioning CD106-positive, short-term hematopoietic stem cells trigger diabetic neuropathy in mice by cell fusion

Miwako Katagi[1], Tomoya Terashima[1], Natsuko Ohashi[1], Yuki Nakae[1], Akane Yamada[1], Takahiko Nakagawa[1,2], Itsuko Miyazawa[3], Hiroshi Maegawa[3], Junko Okano[4], Yoshihisa Suzuki[4], Kazunori Fujino[5], Yutaka Eguchi[5] and Hideto Kojima[1,*]

[1]Department of Stem Cell Biology and Regenerative Medicine, Shiga University of Medical Science, Otsu, Shiga, Japan; [2]Department of Nephrology, Rakuwakai Otowa Hospital, Kyoto, Japan; [3]Department of Internal Medicine, [4]Department of Plastic and Reconstructive Surgery, [5]Department of Critical and Intensive Care Medicine, Shiga University of Medical Science, Otsu, Shiga, Japan

* Address correspondence to: Hideto Kojima, Department of Stem Cell Biology and Regenerative Medicine, Shiga University of Medical Science, Setatsukinowa-cho, Otsu-City, Shiga, 520-2192, JAPAN

Email: kojima@belle.shiga-med.ac.jp

Tel: 81-77-548-2206, Fax: 81-77-548-2642

Supplementary figure 1.

FACS analysis of LSK cells in STZ-induced (Type 1) and high fat diet-induced (Type 2) diabetes model.

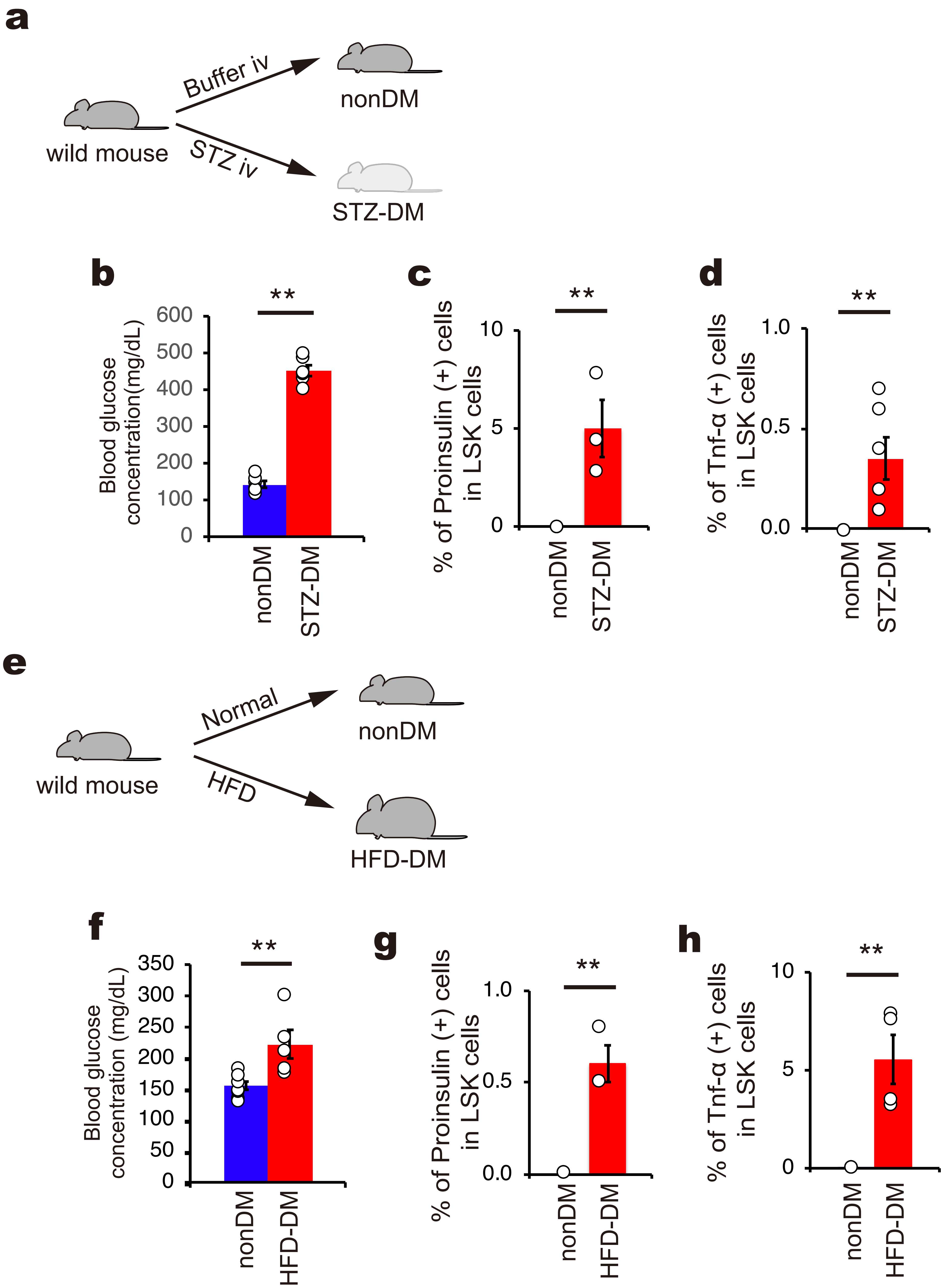

Supplementary figure 1. FACS analysis of LSK cells in STZ-induced (Type 1) and high fat diet-induced (Type 2) diabetes model.

a, A schematic experimental design for the generation of diabetic mice (STZ-DM) and nonDM mice. b, Blood glucose concentration of nonDM and STZ-DM (n = 6 per group). c, FACS analysis of proinsulin-positive cells in the LSK fraction from mononuclear cells in nonDM and STZ-DM. The percentage of proinsulin-positive cells among LSK cells (n = 3 per group). d, FACS analysis of TNF-α-positive cells among LSK cells from mononuclear cell in nonDM and STZ-DM. The percentage of TNF-α-positive cells among LSK cells (n = 6 per group). Data are indicated as means ± SE. **: $P < 0.01$. e, A schematic experimental design for the generation of high fat diet-induced diabetic mice (HFD-DM) and nonDM mice. f, Blood glucose concentration of nonDM (n = 7) and HFD-DM (n = 5). g, FACS analysis of proinsulin-positive cells in the LSK fraction from mononuclear cells in nonDM and HFD-DM. The percentage of proinsulin-positive cells among LSK cells (n = 3 per group). h, FACS analysis of TNF-α-positive cells among LSK cells from mononuclear cell in nonDM and HFD-DM. The percentage of TNF-α-positive cells among LSK cells in nonDM (n = 3) and HFD-DM (n = 5). Data are indicated as means ± SE. **: $P < 0.01$.

Supplementary figure 2.

Cell fusion and development of neuropathy in mice transplanted with total bone marrow cells from diabetic mice.

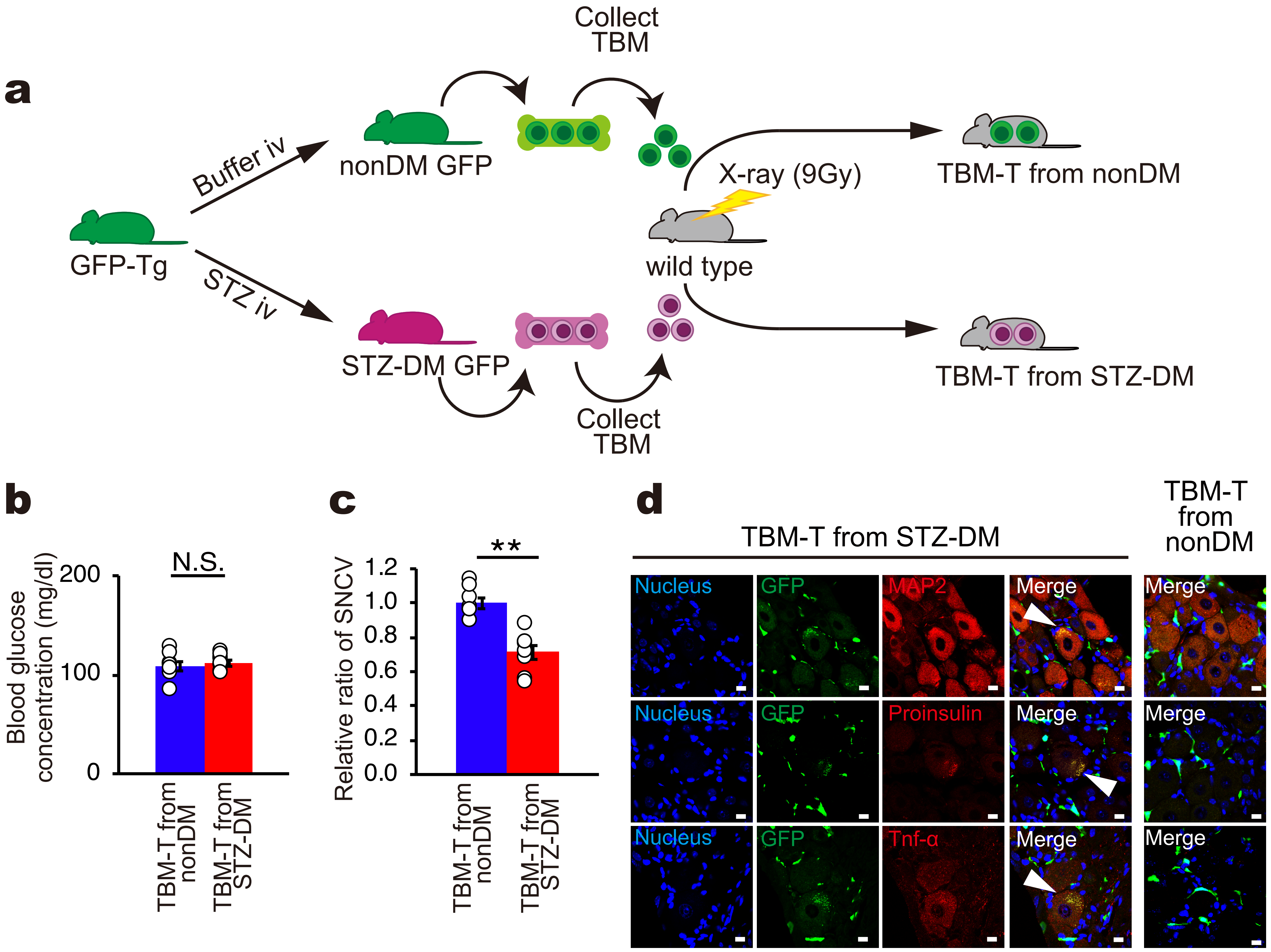

Supplementary figure 2. Cell fusion and development of neuropathy in mice transplanted with total bone marrow cells from diabetic mice.

a, A schematic experimental design for the transplantation of nonDM/STZ-DM total bone marrow cells (TBM) into normoglycemic mice. Diabetes (DM) was induced by STZ in GFP-Tg mice (STZ-DM GFP) while intravenous citrate buffer injection was used for the nonDM controls (nonDM GFP). Three months later, TBM obtained either from nonDM or STZ-DM mice were transplanted into 9Gy lethally-irradiated normoglycemic wild type mice (TBM-T from nonDM or TBM-T from STZ-DM, respectively). b, Blood glucose concentration of TBM-T from nonDM (n = 8) and TBM-T from STZ-DM (n = 8). N.S means not significant. c, Relative ratio of SNCV in the sciatic nerve following TBM-T from STZ-DM mice (n = 8) compared to TBM-T from nonDM donors (n = 8). d, Immunofluorescent staining of the dorsal root ganglion (DRG) showing nuclei (blue), GFP (green), MAP2 (red), proinsulin (red), and TNF-α (red), and merged images in TBM-T from nonDM and TBM-T from STZ-DM mice. Arrowheads showed fusion cells. Scale bars = 10 μm.

Supplementary figure 3. Gene expression of microarray analysis

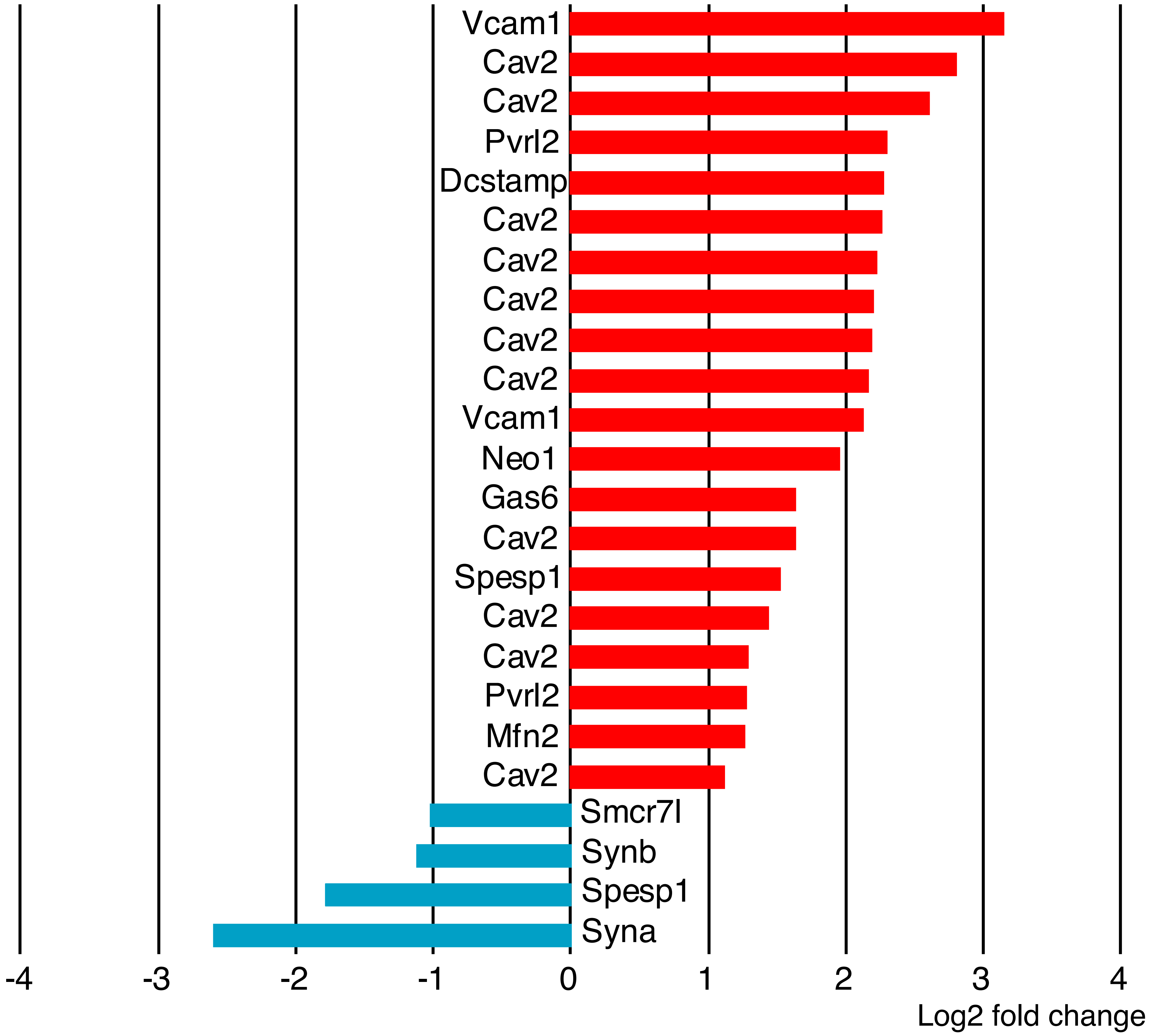

Supplementary figure 3. Gene expression of microarray analysis

Relative gene expression levels in diabetic LSK cells compared with nonDM LSK cells by microarray analysis (Gene Expression Omnibus (GEO) accession number: GSE117088). The red colored bars indicated upregulation of gene expression and the blue colored bars indicated downregulation of gene expression.

Vcam1: vascular cell adhesion molecules 1, Cav2: caveoline 2, Pvrl2: poliovirus receptor-related 2, DcStamp: dendrocyte expressed seven transmembrane protein, Neo1: neogenin, Gas6: growth arrest specific 6, Spesp1: sperm equtatorial segment protein 1, Mfn2: motifusin 2, Smcr7l: Smith-Magenis syndrome chromosome region candidate gene 7 protein-like, Synb: Syncytin b, Syna: Syncytin a

Supplementary figure 4.

CD106- or Tnf-α-positive cells exist in the nonSP fraction of LSK cells in wild mice injected intraperitoneally with 25% glucose.

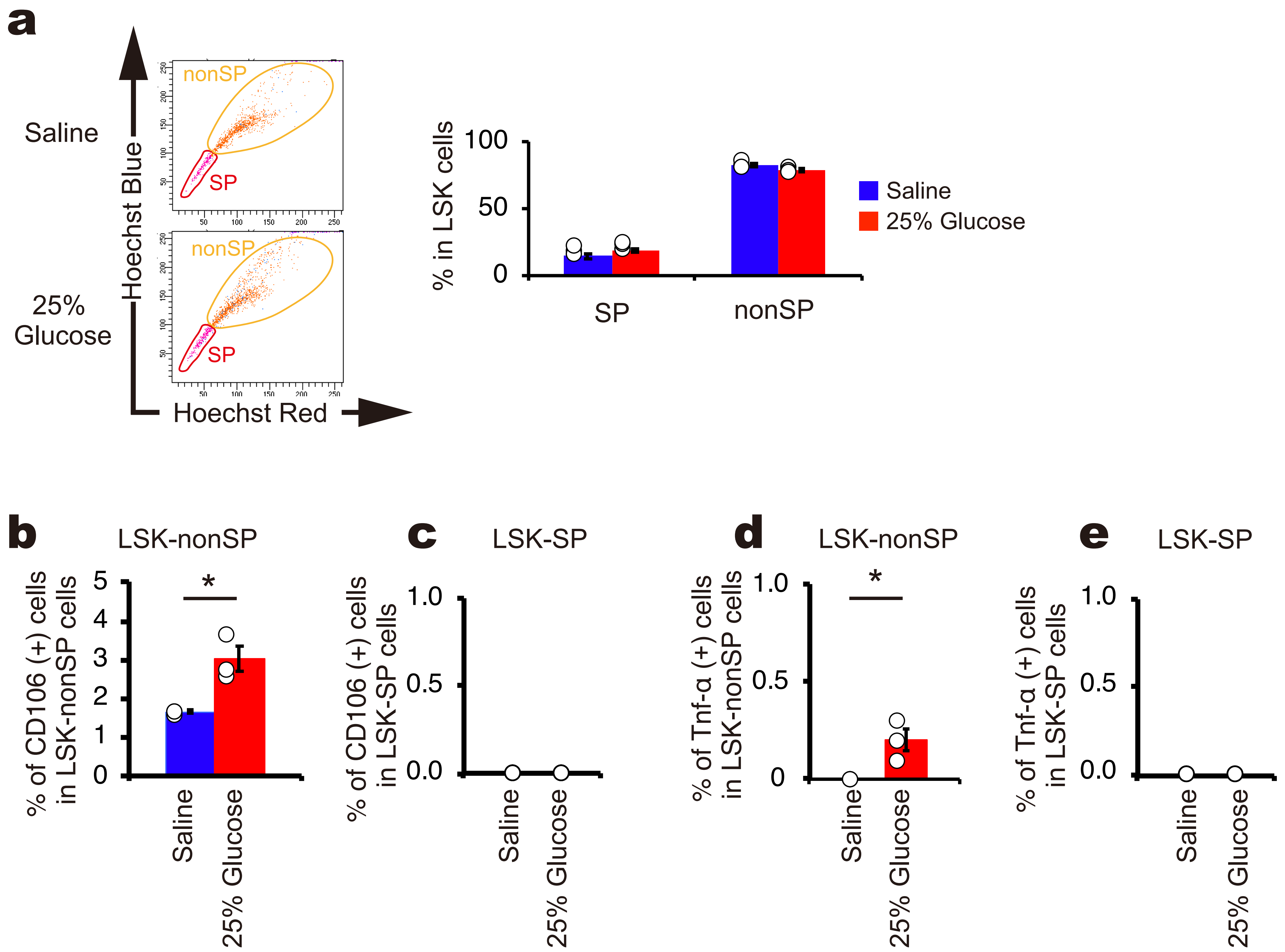

Supplementary figure 4. CD106- or TNF-α-positive cells exist in the nonSP fraction of LSK cells in wild mice injected intraperitoneally with 25% glucose.

a, FACS analysis of SP and/or nonSP fraction in the LSK cells from Saline and 25% Glucose (left panel, SP cells surrounded by red circle, nonSP cells surrounded by orange circle). Percentage of SP fraction or nonSP fraction in LSK cells from mononuclear cells in Saline and 25% Glucose (right panel, n = 3). b, FACS analysis of CD106-positive cells in LSK-nonSP cells from Saline and 25% Glucose. Percentage of CD106-positive cells in LSK-nonSP cells from mononuclear cells in Saline and 25% Gluocse (n = 3). c, FACS analysis of CD106-positive cells in LSK-SP cells from Saline and 25% Glucose. Percentage of CD106-positive cells in LSK-SP cells from mononuclear cells in Saline and 25% Glucose (n = 3). No CD106-positive cells were detected in any of the mice. d, FACS analysis of TNF-α-positive cells in LSK-nonSP cells from Saline and 25% Glucose. Percentage of TNF-α-positive cells in LSK-nonSP from mononuclear cells in Saline and 25% Glucose (n = 3). e, FACS analysis of TNF-α-positive cells in LSK-SP cells from Saline and 25% Glucose. Percentage of TNF-α-positive cells in LSK-SP from mononuclear cells in Saline and 25% Glucose (n = 3). No TNF-α-positive cells were detected in any mice.

Data are indicated as means ± SE. *: $P < 0.05$.

Supplementary figure 5. The full gel images of electrophoresis

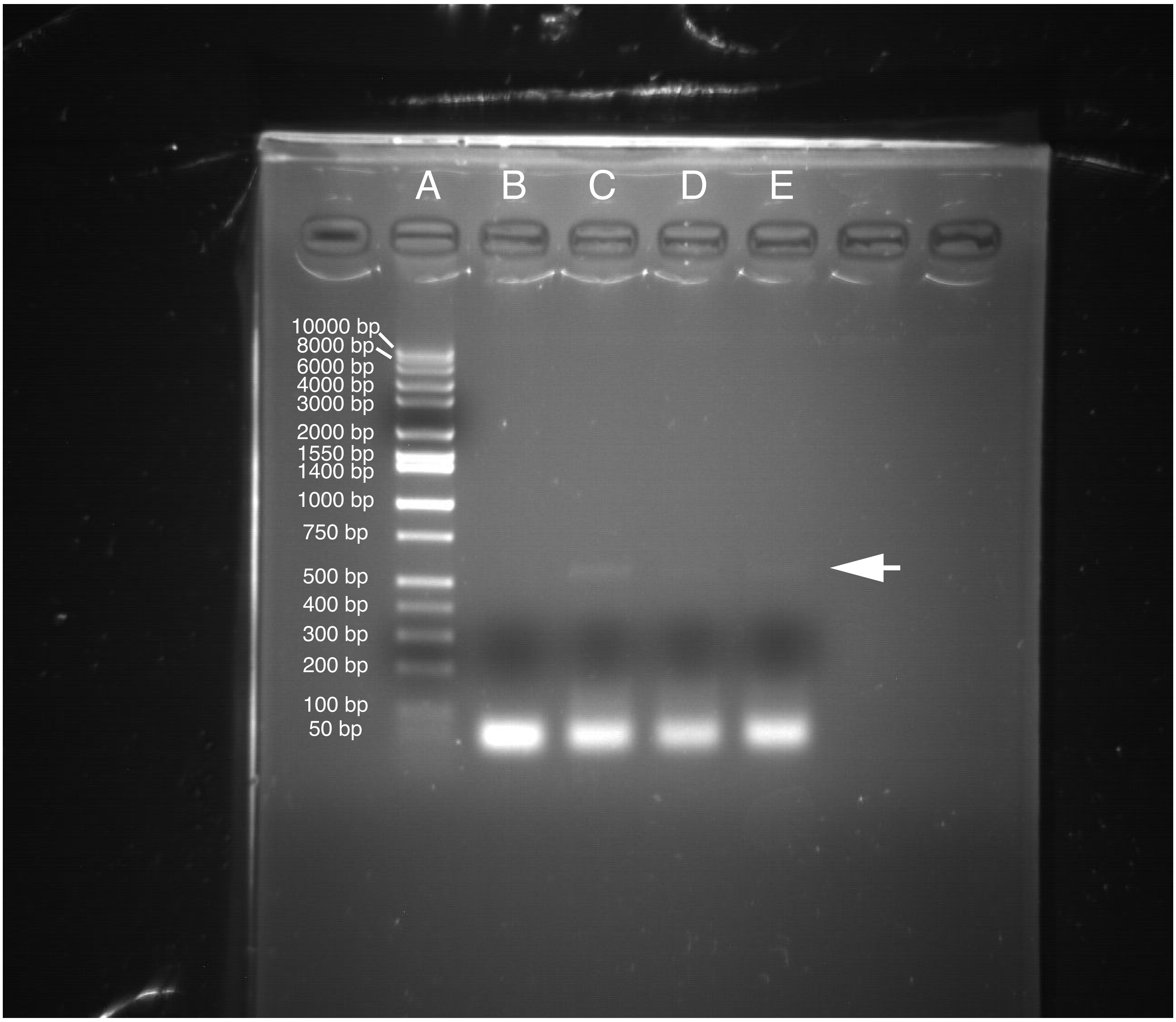

Supplementary figure 5. The full gel images of electrophoresis

This image is the full image of the electrophoresis shown in Figure 1d.

Lane A is the marker (ALL PURPOSE HI-LO DNA Marker, Bionexus, Inc.), Lane B is the PCR product of water as negative control, Lane C is the PCR product of gDNA extracted from the DRG of Ayu1 TBM BMT to lsl-tomato non-DM mice, Lane D is the PCR product of gDNA extracted from the DRG of Ayu1 TBM BMT to lsl-tomato DM mice, and Lane E is the PCR product of gDNA extracted from the liver of Ayu1 TBM BMT to lsl-tomato DM mice. We performed electrophoresis on a 1.5% agarose gel. LaneA to LaneD are used in the Figure 1d.
