# Supplementary material for: Malfunctioning CD106-positive, short-term hematopoietic stem cells trigger diabetic neuropathy in mice by cell fusion
Source: Commun Biol. 2021 May 14;4:575. doi: 10.1038/s42003-021-02082-5 (PMC8121918; doi:10.1038/s42003-021-02082-5)
Supplement: Supplementary file 3 — Description of Additional Supplementary Files [file 42003_2021_2082_MOESM3_ESM.pdf]

Description of Additional Supplementary Files

File name: Supplementary Data 1

Description: All raw data that were graphed.
