# Supplementary material for: Malfunctioning CD106-positive, short-term hematopoietic stem cells trigger diabetic neuropathy in mice by cell fusion
Source: Commun Biol. 2021 May 14;4:575. doi: 10.1038/s42003-021-02082-5 (PMC8121918; doi:10.1038/s42003-021-02082-5)
Supplement: Supplementary file 5 — Reporting Summary [file 42003_2021_2082_MOESM5_ESM.pdf]

nature research

Corresponding author(s): Hideto Kojima

Last updated by author(s): Mar 7, 2021

Reporting Summary

Nature Research wishes to improve the reproducibility of the work that we publish. This form provides structure for consistency and transparency in reporting. For further information on Nature Research policies, see our Editorial Policies and the Editorial Policy Checklist.

Statistics

For all statistical analyses, confirm that the following items are present in the figure legend, table legend, main text, or Methods section.

n/a	Confirmed	
[ ]	[x]	The exact sample size (*n*) for each experimental group/condition, given as a discrete number and unit of measurement
[ ]	[x]	A statement on whether measurements were taken from distinct samples or whether the same sample was measured repeatedly
[ ]	[x]	The statistical test(s) used AND whether they are one- or two-sided<br>*Only common tests should be described solely by name; describe more complex techniques in the Methods section.*
[x]	[ ]	A description of all covariates tested
[x]	[ ]	A description of any assumptions or corrections, such as tests of normality and adjustment for multiple comparisons
[x]	[ ]	A full description of the statistical parameters including central tendency (e.g. means) or other basic estimates (e.g. regression coefficient) AND variation (e.g. standard deviation) or associated estimates of uncertainty (e.g. confidence intervals)
[ ]	[x]	For null hypothesis testing, the test statistic (e.g. *F*, *t*, *r*) with confidence intervals, effect sizes, degrees of freedom and *P* value noted<br>*Give P values as exact values whenever suitable.*
[x]	[ ]	For Bayesian analysis, information on the choice of priors and Markov chain Monte Carlo settings
[ ]	[ ]	For hierarchical and complex designs, identification of the appropriate level for tests and full reporting of outcomes
[x]	[ ]	Estimates of effect sizes (e.g. Cohen's *d*, Pearson's *r*), indicating how they were calculated

Our web collection on statistics for biologists contains articles on many of the points above.

Software and code

Policy information about availability of computer code

Data collection	FACSAria Fusion (Beckton Dickinson), FACS CantoII (Beckton Dickinson), Medelec Sapphire EMG (Medelec), C1si confocal microscope (Nikon), FreeStyle (Nipro)
Data analysis	EZ-C1s software (Nikon), FACSDiva software (Beckton Dickinson), Photoshop CC (Adobe), Illustrator CC (Adobe), Excel (Microsoft),SPSS statistics version22(IBM)

For manuscripts utilizing custom algorithms or software that are central to the research but not yet described in published literature, software must be made available to editors and reviewers. We strongly encourage code deposition in a community repository (e.g. GitHub). See the Nature Research guidelines for submitting code & software for further information.

Data

Policy information about availability of data

All manuscripts must include a data availability statement. This statement should provide the following information, where applicable:
- Accession codes, unique identifiers, or web links for publicly available datasets
- A list of figures that have associated raw data
- A description of any restrictions on data availability

Microarray data (GEO accession number:GSE117088)
we provide the raw data of all figures in supplementary data.

Field-specific reporting

Please select the one below that is the best fit for your research. If you are not sure, read the appropriate sections before making your selection.

- [x] Life sciences
- [ ] Behavioural & social sciences
- [ ] Ecological, evolutionary & environmental sciences

For a reference copy of the document with all sections, see nature.com/documents/nr-reporting-summary-flat.pdf

Life sciences study design

All studies must disclose on these points even when the disclosure is negative.

Sample size	We have used animals for our experiments. Sample sizes were set as minimum as possible to detect the statistical significance in each experiment.
Data exclusions	Before the collection of the data, we set the design that we can obtain fair decision.<br>For that purposes, we exclude animals that shows abnormal condition such as extraordinary low body weight or healthy problems before the data sampling.
Replication	We did not do replicated experiment in all studies.
Randomization	We did randomization of all animals and samples to adjust the study design.
Blinding	Data from each animal or each sample were obtained under blindness and with no experimenter's bias.

Reporting for specific materials, systems and methods

We require information from authors about some types of materials, experimental systems and methods used in many studies. Here, indicate whether each material, system or method listed is relevant to your study. If you are not sure if a list item applies to your research, read the appropriate section before selecting a response.

Materials & experimental systems

n/a	Involved in the study	
	x	Antibodies
x		Eukaryotic cell lines
x		Palaeontology and archaeology
	x	Animals and other organisms
x		Human research participants
x		Clinical data
x		Dual use research of concern

Methods

n/a	Involved in the study	
x		ChIP-seq
	x	Flow cytometry
x		MRI-based neuroimaging

Antibodies

Antibodies used	Biotin Mouse Lineage Panel (BD Pharmingen, Cat#: 559971) including following 5 monoclonal anitbodies: Biotin Hamster anti-Mouse CD3e (clone145-2C11), Biotin Rat anti-Mouse CD45R (clone RA36B2), Biotin Rat anti-Mouse Ly-6G and Ly-6C (clone RB6-8C5), Biotin Rat anti-Mouse CD11b (clone M1/70) Biotin Rat anti-Mouse TER-119/Erythroid cells (clone TER-119).,<br>PE-Cy7 streptavidin (BD Pharmingen, Cat#: 557598),<br>APC Rat Anti-Mouse CD117 (clone 2B8, BD Pharmingen, Cat#: 553356),<br>APC-Cy7 Rat Anti-Mouse Ly-6A/E (clone D7, BD Pharmingen, Cat#: 560654),<br>FITC Rat Anti-Mouse CD106 (clone 429 (MVCAM.A), BD Pharmingen, Cat#: 561678),<br>CD45 Monoclonal Antibody FITC (clone 30-F11, eBioscience, Cat#: 11-0451-81),<br>PE/Cy7 anti-mouse CD45 (clone 30-F11, BioLegend, Cat#: 103113),<br>APC anti-mouse CD105 (clone MJ7/18, BioLegend, Cat#: 120413),<br>TNF alpha Monoclonal Antibody PE (clone MP6-XT22, eBioscience, Cat#: 12-7321-82),<br>Insulin Rabbit mAb (clone C27C9, Cell signaling technology, Cat#: 3014),<br>Anti-rabbit IgG(H+L), F(ab')2 Fragment (PE Conjugate) (Cell signaling technology, Cat#: 8885),<br>MAP2 Antibody (Cell signaling technology, Cat#: 4542S),<br>Anti-TNF alpha antibody (abcam, Cat#: ab6671),<br>Goat anti-Guinea Pig IgG (H+L) Highly Cross-Adsorbed Secondary Antibody, Alexa Fluor 488 (Thermo Fisher Scientific, Cat#: A11073),<br>Donkey anti-Rabbit IgG (H+L) Highly Cross-Adsorbed Secondary Antibody, Alexa Fluor 555 (Thermo Fisher Scientific, Cat#: A31572),<br>Purified anti CD106 antibody (clone 429 (MVCAM.A), Biolegend, Cat#: 105702)
Validation	We decided optimal concentration and reaction time of all antibodies before our study started.

Animals and other organisms

Policy information about studies involving animals; ARRIVE guidelines recommended for reporting animal research

Laboratory animals	C57BL/6J mouse (Japan SLC),<br>C57BL/6-Tg (UBC-GFP) 30Scha/J mice (The Jackson laboratory, Stock #004353),<br>B6.Cg-Gt(ROSA)26Sortm9(CAG-tdTomato)Hze/J (The Jackson laboratory, Stock #007909),<br>Ayu1 promoter-driven Cre recombinase-expressing mice (Niwa H et.al, 1993)
Wild animals	No
Field-collected samples	No
Ethics oversight	No

Note that full information on the approval of the study protocol must also be provided in the manuscript.

Flow Cytometry

Plots

Confirm that:

- [x] The axis labels state the marker and fluorochrome used (e.g. CD4-FITC).
- [x] The axis scales are clearly visible. Include numbers along axes only for bottom left plot of group (a 'group' is an analysis of identical markers).
- [x] All plots are contour plots with outliers or pseudocolor plots.
- [x] A numerical value for number of cells or percentage (with statistics) is provided.

Methodology

Sample preparation	Mice were anesthetized and perfused with cold PBS (-) to remove peripheral blood.<br>Tibiae, fumeri and fibulae were taken from perfused mice on ice.<br>Total bone marrow were collected from those bones.<br>Finally, we isolate mononuclear cells from total bone marrow by Ficoll-Paque PLUS.<br>Following antibody staining steps were described in "Methods" section in our manuscript.
Instrument	FACSAriaFusion, FACS CantoII
Software	FACSDiva software
Cell population abundance	We did it
Gating strategy	Gating strategy is described in "Method" section in our manuscript.

- [ ] Tick this box to confirm that a figure exemplifying the gating strategy is provided in the Supplementary Information.
